# Supplementary material for: Fermi surface chirality induced in a TaSe2 monosheet formed by a Ta/Bi2Se3 interface reaction
Source: Nat Commun. 2022 May 5;13:2472. doi: 10.1038/s41467-022-30093-1 (PMC9072342; doi:10.1038/s41467-022-30093-1)
Supplement: Supplementary file 1 — Supplementary Information [file 41467_2022_30093_MOESM1_ESM.pdf]

Supplementary Information for ”Fermi surface chirality induced in a $TaSe_2$ monosheet formed by a Ta/Bi_2Se_3 interface reaction”

Andrey Polyakov[1], Katayoon Mohseni[1], Roberto Felici[2], Christian Tusche[3,4], Ying-Jun Chen[3,4], Vitaly Feyer[3,4], Jochen Geck[5,6], Tobias Ritschel[5], Arthur Ernst[7], Juan Rubio-Zuazo[8], German R. Castro[8], Holger L. Meyerheim[1,*], and Stuart S. P. Parkin[1]

[1]Max-Planck-Institut für Mikrostukturphysik, Weinberg 2, 06120 Halle (Germany)
[2]Consiglio Nazionale delle Ricerche - SPIN, Via del Politecnico, 1 Roma 00133, (Italy)
[3]Forschungszentrum Jülich GmbH, Peter Grünberg Institut (PGI-6), 52425 Jülich, Germany
[4]Fakultät für Physik, Universität Duisburg-Essen, 47057 Duisburg, Germany
[5]Institut für Festkörper- und Materialphysik, Technische Universität Dresden, 01062 Dresden (Germany)
[6]Würzburg-Dresden Cluster of Excellence ct.qmat, Technische Universität Dresden, 01062 Dresden (Germany)
[7]Institut für Theoretische Physik, Johannes Kepler Universität, A 4040 Linz, Austria
[8]SpLine, Spanish CRG BM25 Beamline at the ESRF (The European Synchrotron), F-38000 Grenoble, France
[*]holger.meyerheim@mpi-halle.mpg.de

1 A. X-Ray Diffraction

1.1 (a) Data for 1H-$TaSe_2$

Integrated intensities of the 1H-$TaSe_2$ monosheet were collected as a given detector position calculated for the reflection (hkℓ) by rotating the sample around the surface normal. Fig. 1 shows an example for the (10ℓ) reflections for ℓ in the range between ℓ=3 and 8. The integrated intensities were used for the structure analysis as outlined by Fig.3 of the main text.

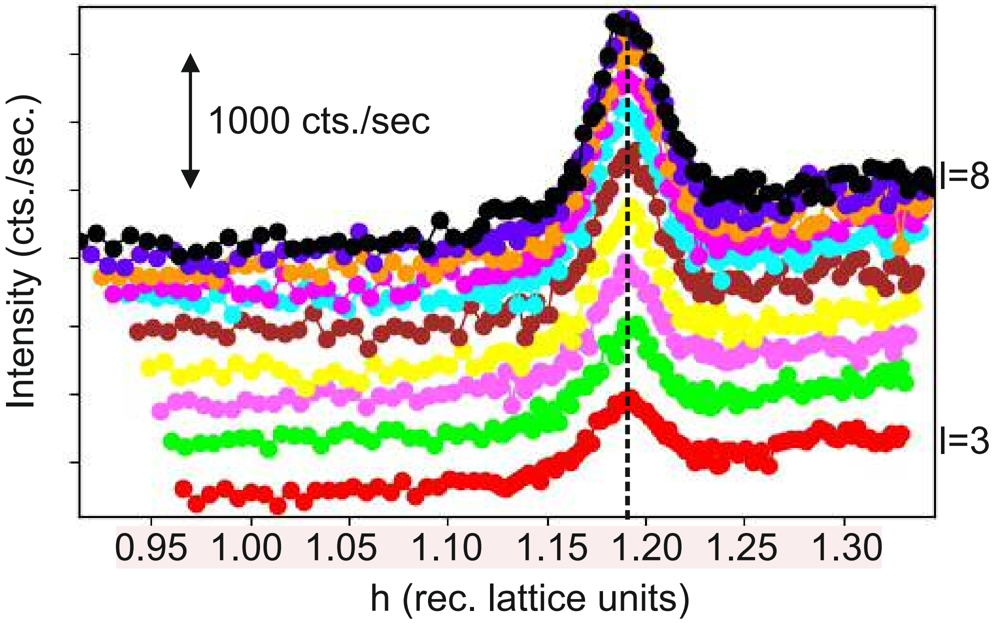

Figure 1. Reflection intensity collected for 1H-$TaSe_2$ versus a^* in reciprocal space. Reflections are shown for values of ℓ between 3 and 8 in steps of $\Delta\ell$=0.5 reciprocal lattice units. The peak position is at h=1.19 reciprocal lattice units. Coordinates are referred to the Bi_2Se_3(0001) substrate (a=b=4.14 Å, c=28.65 Å. Integrated intensities were used for the structure analysis as outlined in Fig.3 of the main text. Curves are shifted for clarity.

1.2 (b) Intensity distribution for 2H-$TaSe_2$

The experimental integrated intensities were also analyzed by consideration of thicker films. One example is shown in Fig. 2 for the model of a 2H-type structure, i.e. by considering a second Se-Ta-Se monosheet above the first one. It can be seen directly that the calculated intensities do not fit the experimental ones.

The addition of a second Se-Ta-Se triple-layer (TL) in the calculation leads to the appearance of a second maximum resulting from the interference between the two TL's. By comparison with the experimental data, the 2H-$TaSe_2$ can clearly excluded.

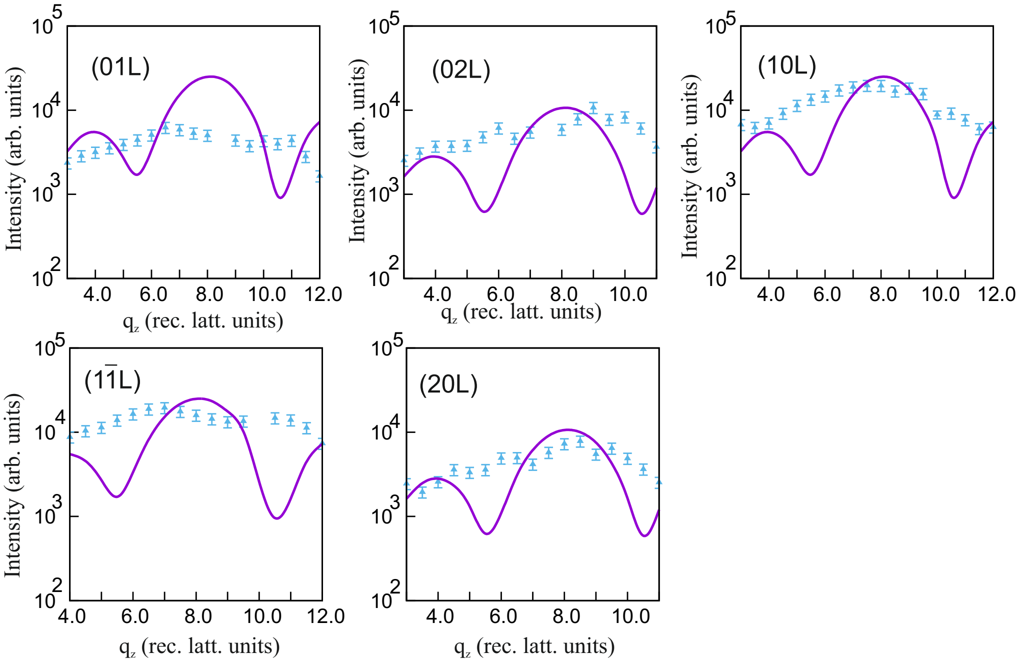

Figure 2. Experimental (symbols) and calculated (lines) intensities for for $TaSe_2$/Bi2Se3(0001). The calculation is based on a structure model corresponding to a complete 2H unit cell i.e. involving a second Se-Ta-Se triple-layer. This induces the appearance of a minimum between two main maxima (Bragg-reflections of the two unit cell structure) in clear disagreement with the experimental data.

2 B. Photoemission

2.1 (a) Electron Energy Distribution of the H-$TaSe_2$ monosheet

Fig. 3 shows the electron energy distribution curves along the $\overline{\Gamma}$-$\overline{K}$ and the $\overline{\Gamma}$-$\overline{M}$ direction derived from the band structure shown in Fig. 5(a) of the main text. In general, the low-lying strongly dispersive states are related to Ta-5d states which cross the Fermi level (E_F) between $\overline{M}$ and $\overline{\Gamma}$. Thus, the H-type monosheet is metallic. Deeper lying dispersing states are related to Se-p-states.

3 C. Density functional theory

3.1 (a) Calculation of the vertical relaxation of the Ta atom

Since the vertical position of Ta atoms is decisive for the observed in-plane spin-texture, we have calculated the total energy of the interface structure as a function of the Ta vertical position (z) within the hexagonal coordination prism. To this end we used a structural model consisting of a slab of nine quintuple layers (QL) of Bi_2Se_3 and six QL of empty spheres, the latter to simulate vacuum. A single H-$TaSe_2$ triple layer (TL) with in-plane lattice parameters being commensurate with the in-plane lattice parameter of the Bi_2Se_3 substrate (a=b=414 pm) was placed on the substrate. A schematic of the interface between the topmost QL of the Bi_2Se_3 substrate and the $TaSe_2$ monosheet is given in the inset of Fig. 4. The Se-Se distance across the vdW gap at the $TaSe_2$/Bi_2Se_3 interface was derived by total energy minimization to be equal to 335 pm, which is only slightly smaller than the Se-Se distance between neighboring QL's in bulk Bi_2Se_3 (341 pm). The height of the $TaSe_6$ prism was fixed to 337 pm, the experimentally derived value

In the following only the relative vertical Ta coordinate (z) was varied. The results of the calculation is shown in Fig. 4. The total energy as a function of the relative z(Ta) coordinate has two minima, at z(Ta)$\approx$0.42 (global minimum) and at z(Ta)$\approx$0.59 (local minimum).

Thus, the calculations confirm quite well the results of the X-ray structure analysis despite the fact that the assumed $TaSe_2$ structure was assumed to be commensurable rather than incommensurate, which is a requirement to carry out the calculations including the substrate. The Ta vertical shift is a manifestation of the symmetry breaking. The $TaSe_2$ monosheet experiences a polar environment characterized by the presence of the Bi_2Se_3 substrate below and the vacuum above it. From photoemssion

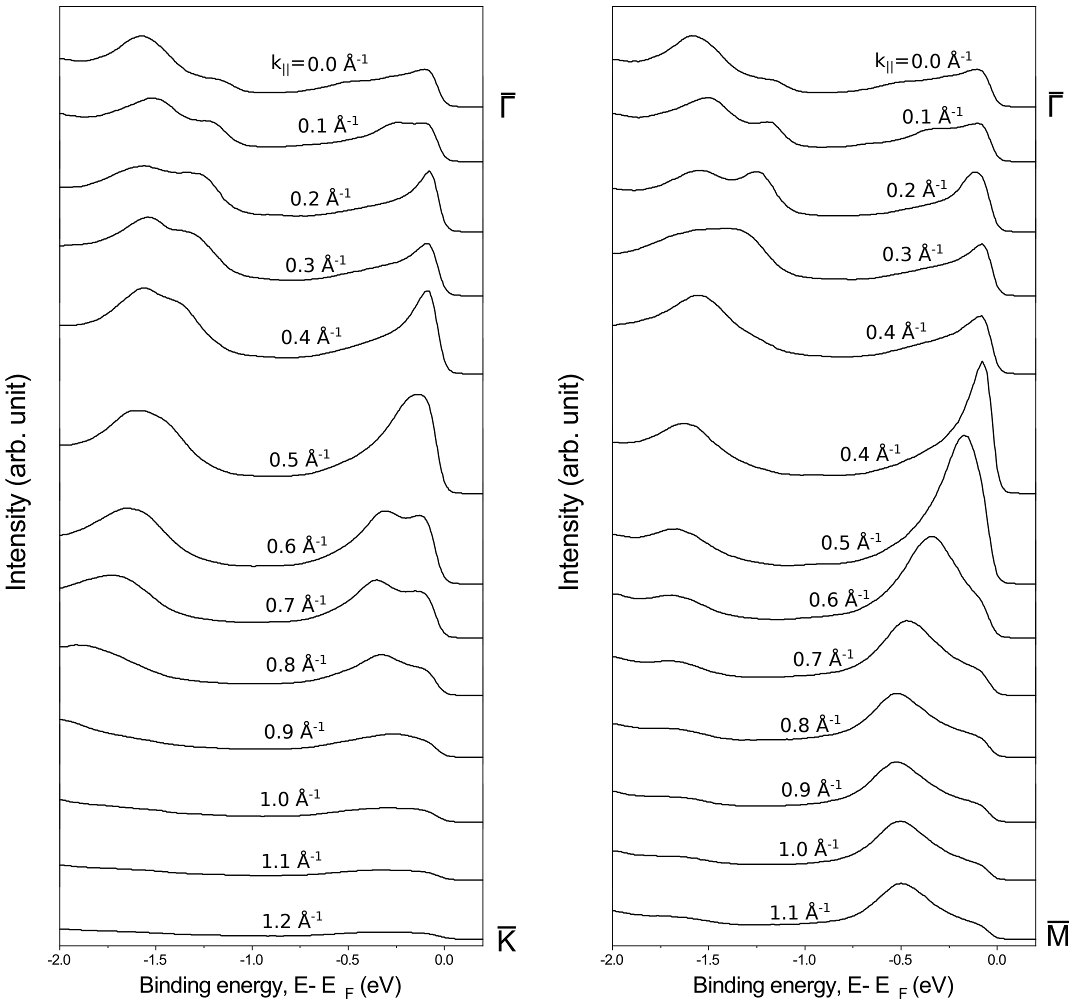

Figure 3. Photoelectron Energy Distribution curves (EDC) for $TaSe_2/Bi_2Se_3(0001)$ derived from the spin-averaged band structure shown in Fig.5(a) of the main text along the $\overline{\Gamma}$-$\overline{K}$ and the $\overline{\Gamma}$-$\overline{M}$ direction.

(see Fig. 5 of the main text) is known that the monosheet is metallic and charge is redistributed to the direction away from the vacuum to the -z direction which makes the z=0.5 position energetically unstable and consequently favors a stronger bond by a downward shift of the Ta atom. In this way also the presence of a local energetic minimum at z=0.59 can be understood as the bond strength to the upper Se atoms is lower due to the lower charge density.

3.2 (b) Fermi Surface for 1H $TaSe_2$ in-plane / out of plane

In order to illustrate the three-dimensional character of the k-space spin texture and its dependency on the z-position of the central Ta atom we show in Fig. 5 a three-dimensional image of the spin-polarization. A color-coded illustration of the x, y and z-component of the spin-polarization is given in Fig. 6. In both images different z-positions of the central Ta atom within the unit cell are considered (z=0.43 nd 0.50). Non-zero x and y-components only occur if the position of the Ta atom is away from the high symmetry point at z=0.5. By contrast, the spin polarization of the z-component is always nonzero and strongest for z=0.5 as expected for the Ising spin texture.

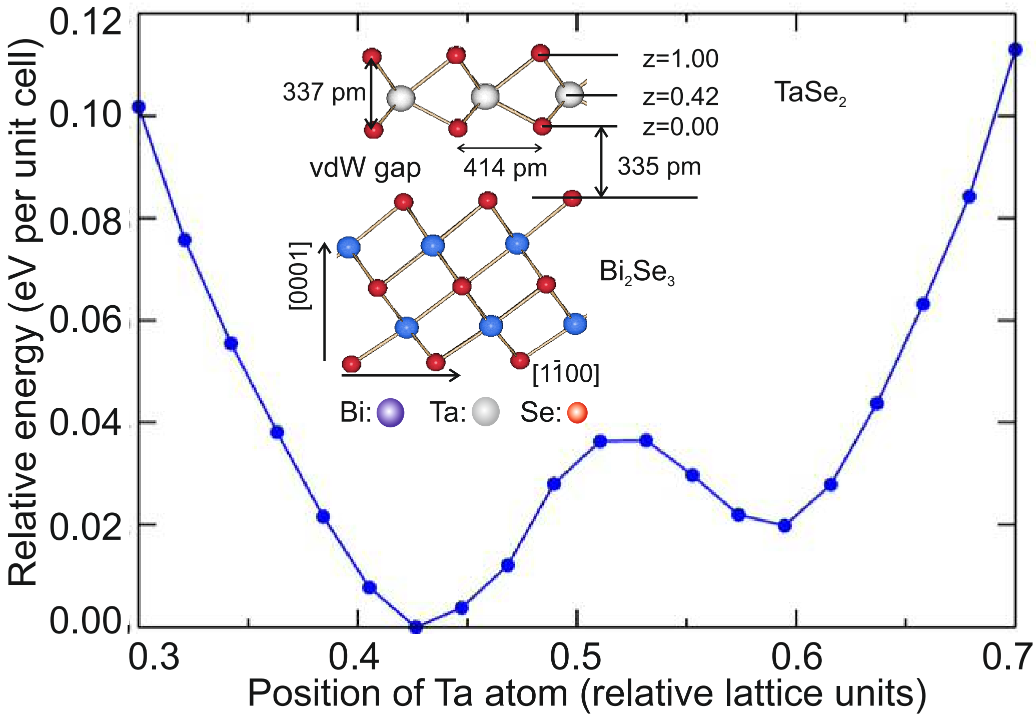

Figure 4. Calculated (relative) total energy of the $TaSe_2/Bi_2Se_3$ structure versus z, the relative vertical coordinate of the Ta atom within the coordination polyhedron (see also Fig.4 of the main text). The global minimum is calculated to be $z \approx 0.42$ in close agreement with experiment. The inset shows the interface structure considered for the calculation.

3.3 (c) Fermi Surface Calculated for 1H $TaSe_2$ K-points

In Fig. 6 of the main text we show that the shape of the Fermi surface feature around the K-point of the 2D Brillouin zone strongly depends on the z-position of the central Ta atom and that the agreement with the photoemission data is at an optimum for z=0.43 in accordance with the x-ray diffraction analysis. In 7 we show the spin-polarization along all three components (x,y and z) focusing on the feature around the K-point and for different Ta z-positions.

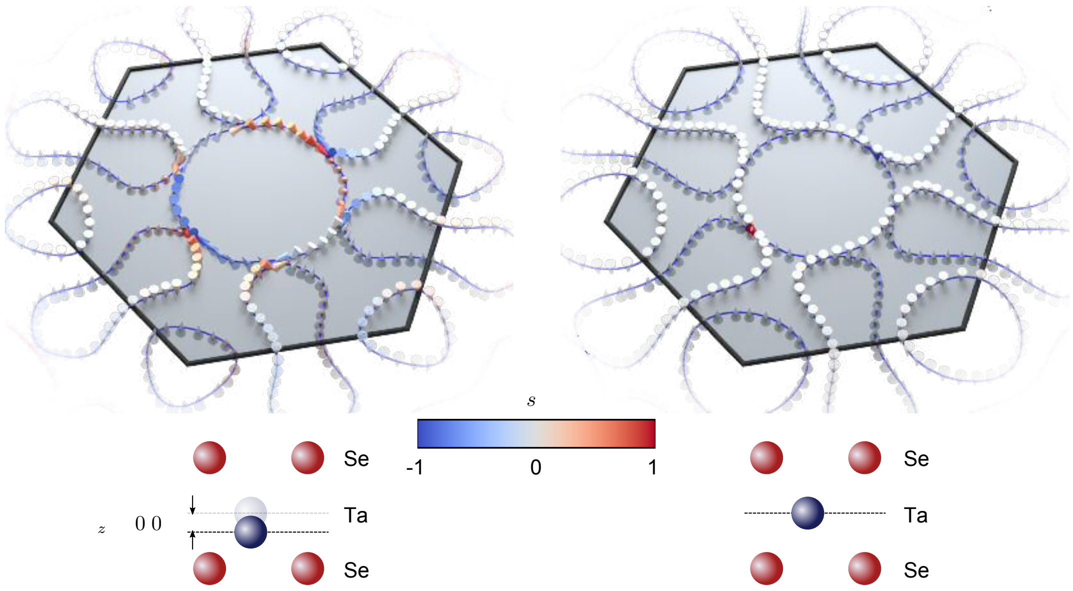

Figure 5. Three-dimensional image of the calculated spin polarization at the Fermi level for 1H-$TaSe_2$ for different z-positions of the central (prismatically) coordinated Ta atom.

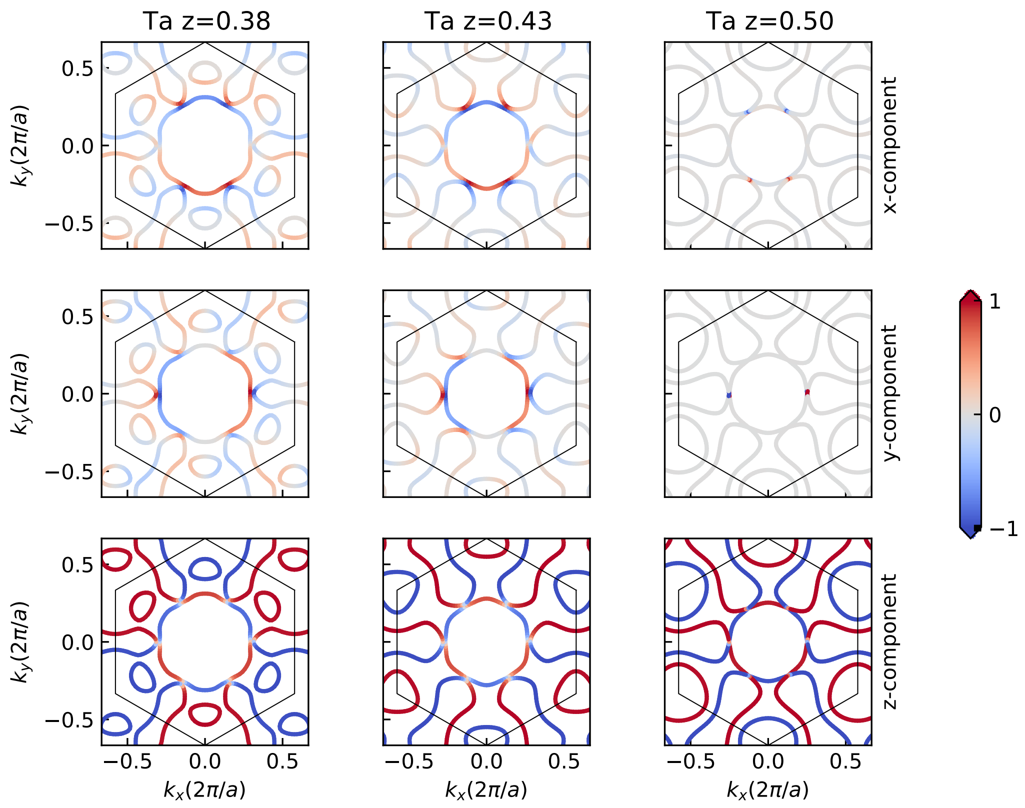

Figure 6. Calculated spin polarization along x,y, and z at the Fermi level for 1H-$TaSe_2$ for different z-positions of the central (prismatically) coordinated Ta atom.

3.4 (d) Fermi Surface for 1T $TaSe_2$

For comparison with the results displayed in Figs. 6 and 7 of the main text for H-$TaSe_2$, we have also calculated the Fermi surface contour and the spin-polarization at E_F for a single sheet of 1T-$TaSe_2$ for different z-position of the central (octahedrally

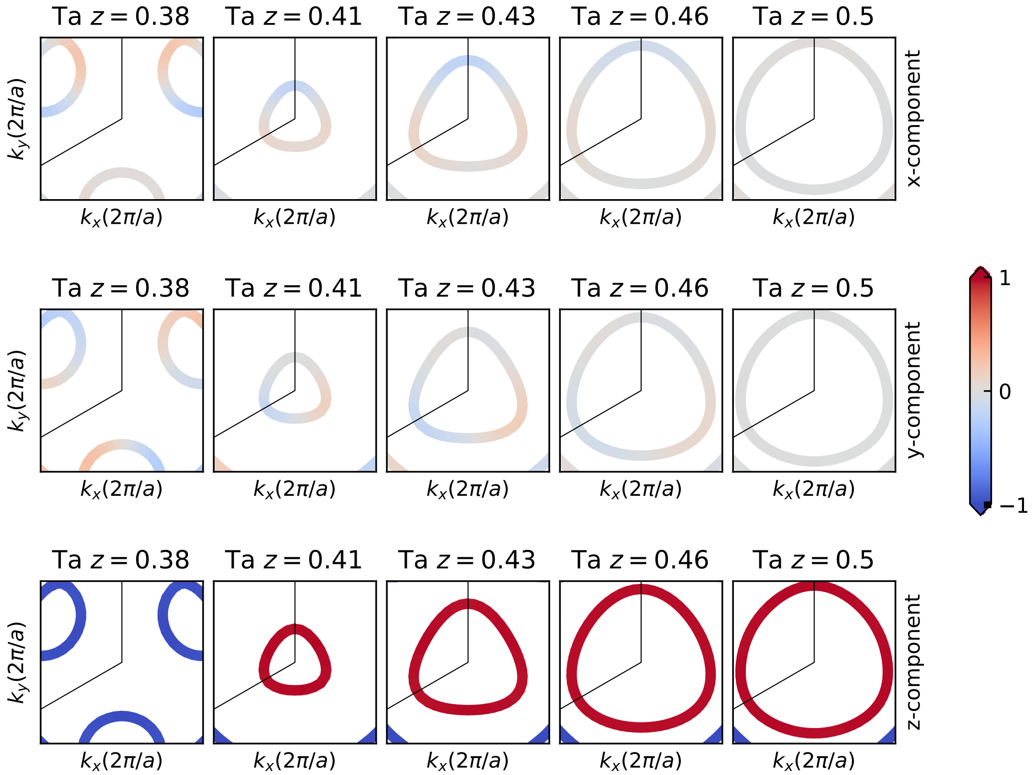

Figure 7. Calculated x-,y, and z-component of the spin polarization at the Fermi level for a monosheet of 1H-$TaSe_2$ for different z-positions of the central (prismatically) coordinated Ta atom as is in Fig. 6, here focusing on the region around the K-point of the Brillouin zone.

coordinated) tantalum. This is shown in Fig. 8. The topology of the Fermi surface does not at all fit to the experimental data shown in Figs. 6 and 7 of the main text.

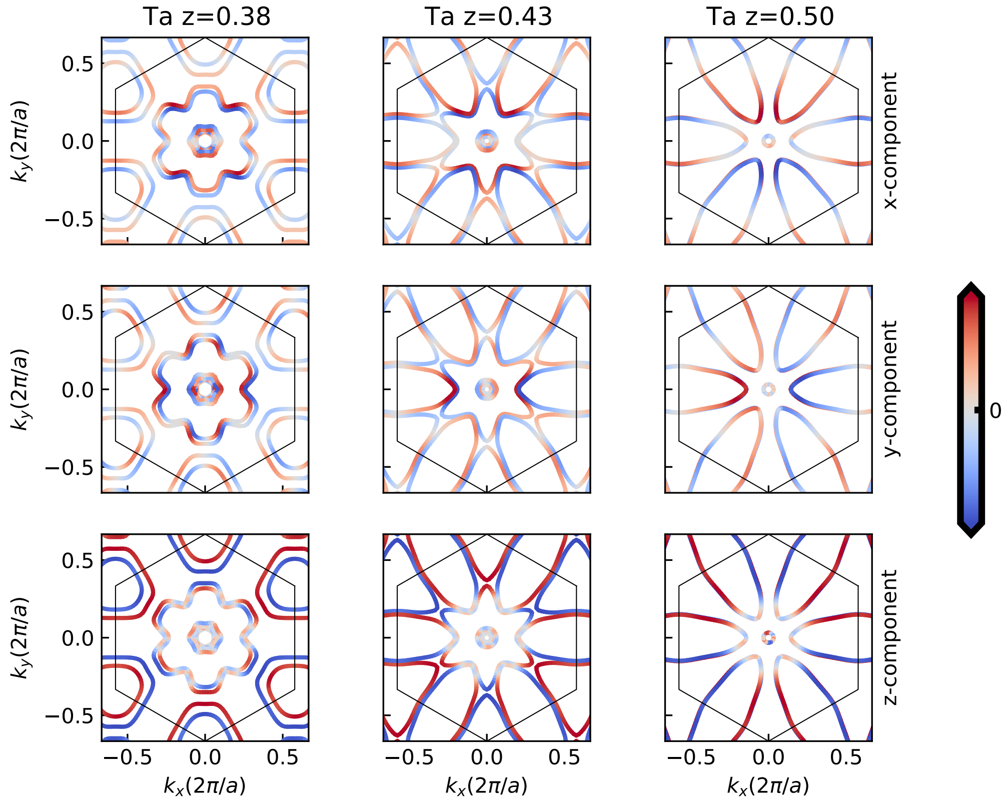

Figure 8. Calculated spin-resolved momentum map at E_F for 1T-$TaSe_2$ for different z-positions of the central octahedrally coordinated Ta atom. Comparison with experiment (see Fig. 6 and 7 of the main text) indicates distinct differences which directly excludes the 1T structure.

3.5 (d) Antiparallel chirality between H-$TaSe_2$ and Bi_2Se_3 states

To confirm the experimental results concerning the spin polarization in H-$TaSe_2$ on Bi_2Se_3 (see e.g. Fig. 5c and 7a of the main text) indicating an antiparallel chirality of the tantalum d-states around the Γ point and the topological surface state (TSS) we have carried out first-principles calculations using self-consistent full relativistic Green function method designed for bulk and semi-infinite systems such as surfaces and interfaces [1–3]. The calculation were carried out within the density functional theory in a generalized gradient approximation [4].

For our study we used the same structural model as outlined in section (a) and shown in Fig. 4. The three panels in Fig. 9 show the band structure along the $\overline{M}$ -$\overline{\Gamma}$ - $\overline{M}$ direction for all components of the spin polarization (x, y, z). Red and blue lines correspond to positive and negative spin polarization, respectively. The simulation reproduces the experimental observations. First of all, the Dirac cone is not strongly affected by the presence of the $TaSe_2$ d-states. The states below and above E_F are associated with the first QL of Bi_2Se_3. They are also spin polarized, mainly due to the interface effects. Most importantly, the calculated spin polarization of the surface states is in qualitative agreement with the the experimental results (see e.g. Fig. 5b and 7a of the main text), namely the antiparallel orientation between the spin texture of the $TaSe_2$ states and the TSS. The antiparallel chirality of the $TaSe_2$ states and the TSS originates from the minimization of the exchange correlation potential, i.e. due to the the Pauli exclusion principle.

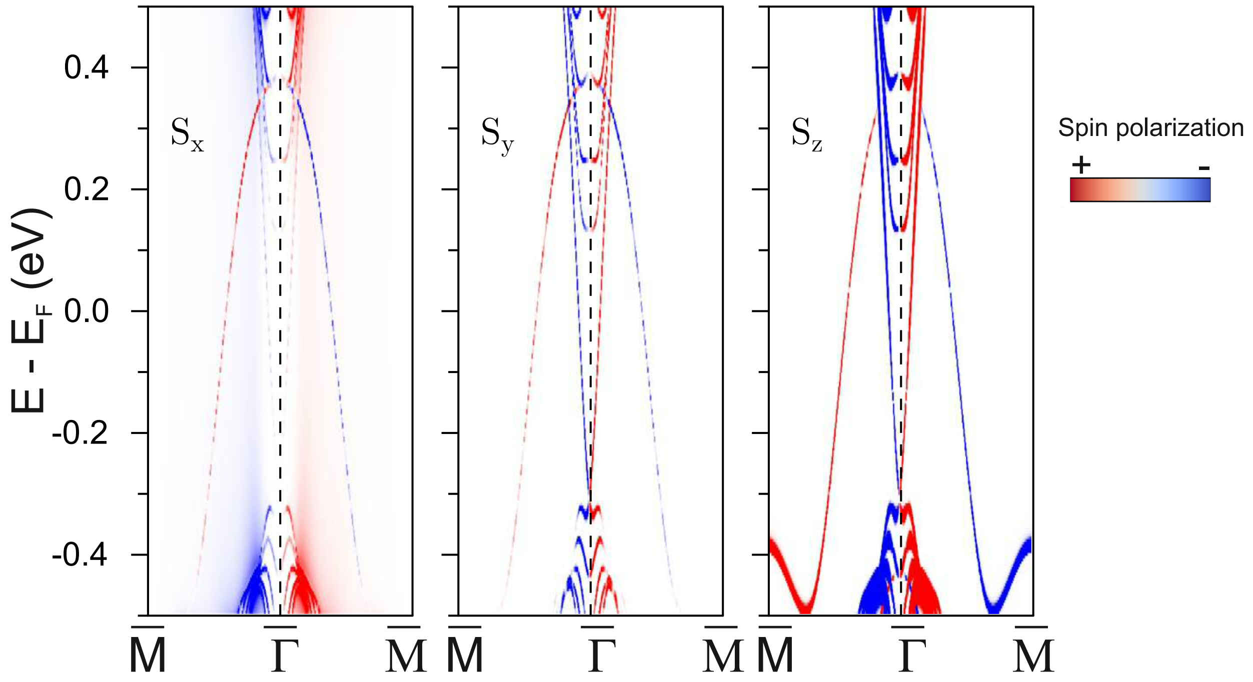

Figure 9. Calculated band structure of a monosheet of $TaSe_2$ on Bi_2Se_3(0001) showing the x-, y, and z-component of the spin polarization. Note, the antiparallel chirality between the TSS of Bi_2Se_3 and the tantalum d-states in qualitative agreement with experiment (see e.g. Figs. 5c and 7a of the main text).

4 References

(1) Lüders, M.; Ernst, A.; Temmerman, W. M.; Szotek, Z.; Durham, P. J. Ab initio angleresolved photoemission in multiple-scattering formulation. Journal of Physics: *Condensed Matter* **2001**, 13, 8587–8606.

(2) Geilhufe, M.; Achilles, S.; Köbis, M. A.; Arnold, M.; Mertig, I.; Hergert, W.; Ernst, A. Numerical solution of the relativistic single-site scattering problem for the Coulomb and the Mathieu potential. *Journal of Physics:* Condensed Matter **2015**, 27, 435202.

(3) Hoffmann, M.; Ernst, A.; Hergert, W.; Antonov, V. N.; Adeagbo, W. A.; Geilhufe, R. M.; Ben Hamed, H. Magnetic and Electronic Properties of Complex Oxides from First-Principles. *Physica Status Solidi (b)* **2020**, 257, 1900671.

(4) Perdew, J. P.; Burke, K.; Ernzerhof, M. Generalized Gradient Approximation Made Simple. *Phys. Rev. Lett.* **1996**, 77, 3865–3868.
